# Influenza A(H5N1) shedding in air corresponds to transmissibility in mammals

**DOI:** 10.1038/s41564-024-01885-6

**Published:** 2024-12-02

**Authors:** Ilona I. Tosheva, Fabien Filaire, Willemijn F. Rijnink, Dennis de Meulder, Bianca van Kekem, Theo M. Bestebroer, Mathis Funk, Monique I. Spronken, C. Joaquin Cáceres, Daniel R. Perez, Mathilde Richard, Marion P. G. Koopmans, Pieter L. A. Fraaij, Ron A. M. Fouchier, Sander Herfst

**Affiliations:** 1 Department of Viroscience, Erasmus University Medical Centre, Rotterdam, the Netherlands; 2 Department of Population Health, College of Veterinary Medicine, University of Georgia, Athens, GA USA; 3 Pandemic and Disaster Preparedness Center, Delft, Rotterdam, the Netherlands; 4 Department of Paediatrics, Erasmus University Medical Center, Rotterdam, the Netherlands

**Keywords:** Influenza virus, Viral transmission, Influenza virus

## Abstract

An increase in spillover events of highly pathogenic avian influenza A(H5N1) viruses to mammals suggests selection of viruses that transmit well in mammals. Here we use air-sampling devices to continuously sample infectious influenza viruses expelled by experimentally infected ferrets. The resulting quantitative virus shedding kinetics data resembled ferret-to-ferret transmission studies and indicated that the absence of transmission observed for earlier A(H5N1) viruses was due to a lack of infectious virus shedding in the air, rather than the absence of necessary mammalian adaptation mutations. Whereas infectious human A(H1N1_pdm_) virus was efficiently shed in the air, infectious 2005 zoonotic and 2024 bovine A(H5N1) viruses were not detected in the air. By contrast, shedding of infectious virus was observed for 1 out of 4 ferrets infected with a 2022 European polecat A(H5N1) virus and a 2024 A(H5N1) virus isolated from a dairy farm worker.

## Abstract (web summary)

Spillover of avian A(H5N1) influenza virus to mammals may favour adaptation to these new hosts. Air sampling of infected ferrets shows that recent A(H5N1) strains, including one from a human case linked to the US cattle outbreak, are efficiently expelled into the air.

## Main

Highly pathogenic avian influenza (HPAI) A(H5) viruses of the A/goose/Guangdong/1/1996 lineage were detected in southern China and have since evolved and spread globally, triggering outbreaks in Europe, Africa, the Americas and Antarctica. Recently, large outbreaks of HPAI A(H5) viruses have been reported in mammals, including farmed minks, foxes and raccoon dogs as well as in sea lions and elephant seals^1–4^. In March 2024, after reports of unexplained symptoms in US dairy cattle, HPAI A(H5N1) virus was isolated from cow samples. This event marked the beginning of a still ongoing epizootic that, as of 5 November 2024, resulted in 442 outbreaks in dairy cattle in 15 US states^5^. This spillover of HPAI A(H5) to cattle is an abrupt change in epidemiology, leading to sustained mammal-to-mammal transmission and an increased risk of exposure for farm workers and various wild and domestic animals, potentially elevating global public health risks. So far, 44 mild human cases of HPAI A(H5N1), mostly presenting as conjunctivitis, have been reported in the USA since April. Twenty-four were linked to infected dairy cows and 19 to poultry, and 1 had no known exposure source^6^.

To assess the public health risk of emerging A(H5N1) influenza viruses, studies commonly use ferret transmission set-ups that only allow transmission via the air from donor to recipient animals. Although this experimental set-up provides important knowledge on the transmission potential, no information on virus shedding kinetics in the air is obtained. Consequently, longstanding questions remain about whether avian influenza viruses fail to transmit between mammals owing to the lack of infectious virus shedding in the air or the absence of necessary mammalian adaptation markers required to start an infection in a new host.

**Fig. 1:**
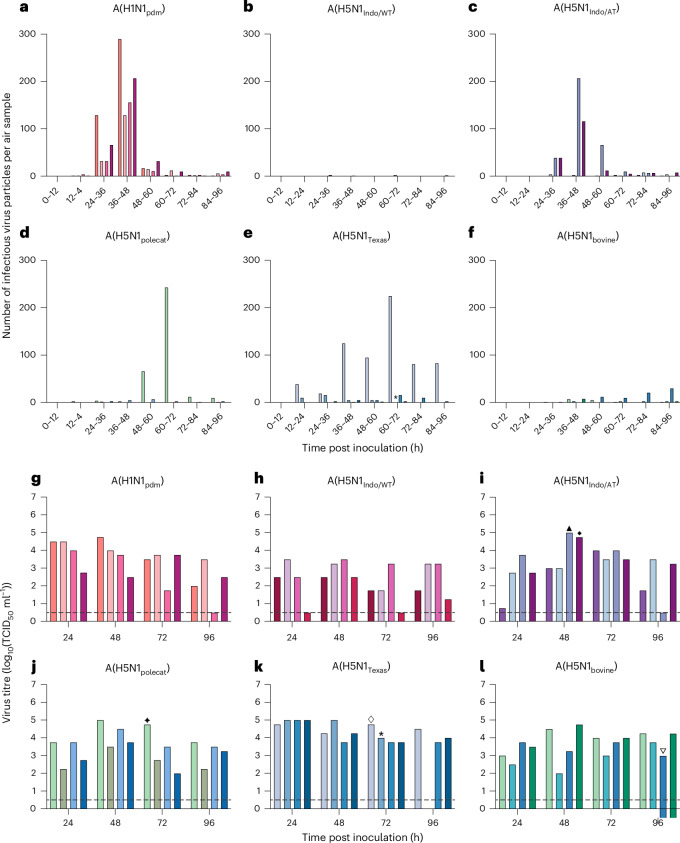
Infectious virus in air samples and ferret nose swabs. **a**–**f**, Infectious virus particles per air sample: A(H1N1_pdm_) (**a**), A(H5N1_Indo/WT_) (**b**), A(H5N1_Indo/AT_) (**c**), A(H5N1_polecat_) (**d**), A(H5N1_Texas_) (**e**) and A(H5N1_bovine_) (**f**). **g**–**l**, Infectious virus titres in ferret nose swabs: A(H1N1_pdm_) (**g**), A(H5N1_Indo/WT_) (**h**), A(H5N1_Indo/AT_) (**i**), A(H5N1_polecat_) (**j**), A(H5N1_Texas_) (**k**) and A(H5N1_bovine_) (**l**). Each differently coloured bar represents a single ferret (four ferrets per virus). The dotted horizontal lines represent the detection limit. The symbols above bars indicate the day of peak virus shedding in the air for A(H5N1)-inoculated animals. *This animal was euthanized at 72 hpi because of reaching predetermined humane endpoints; thus, no nose swabs were collected after the 72 hpi timepoint. Source data

**Extended Data Fig. 1:**
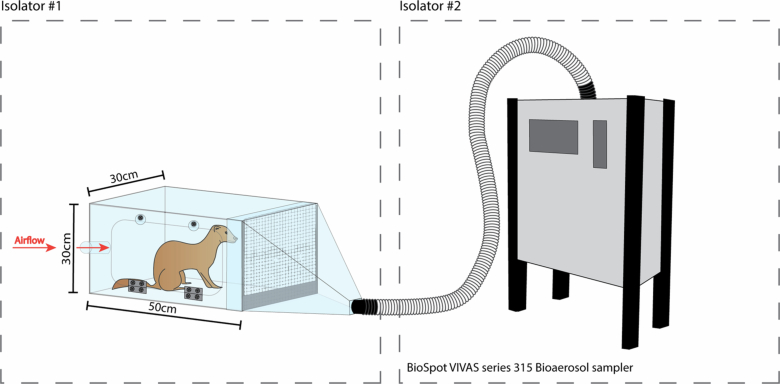
Experimental air-sampling set-up. For the collection of air-samples, the BioSpot-VIVASTM Series 315 bioaerosol sampler (Aerosol Devices, USA) was directly connected to a ferret cage holding an individually housed ferret, via a 1.4 m long tubing. The BioSpot was operated with a flowrate of 15 L min-1 under the following conditions: conditioner: 5 °C; initiator: 45 °C; moderator: 18 °C; nozzle: 27 °C; sample holder: between 13-16 °C. No extra airflow was supplied to the ferret cage, in addition to the 15L min-1 applied by the BioSpot sampler. With a cage volume of 45 L (30 ×30 x 50 cm) and a flow rate of 900L h-1 (60 x 15L min-1), this results in 20 (900L h-1 / 45 L) air changes per hour. The ferret cage and BioSpot were housed in separate, interconnected, negatively pressurized class 3 isolators. This separation was necessary because the BioSpot generated excessive heat, which led to an increased ambient temperature within the isolator.

**Extended Data Fig. 2:**
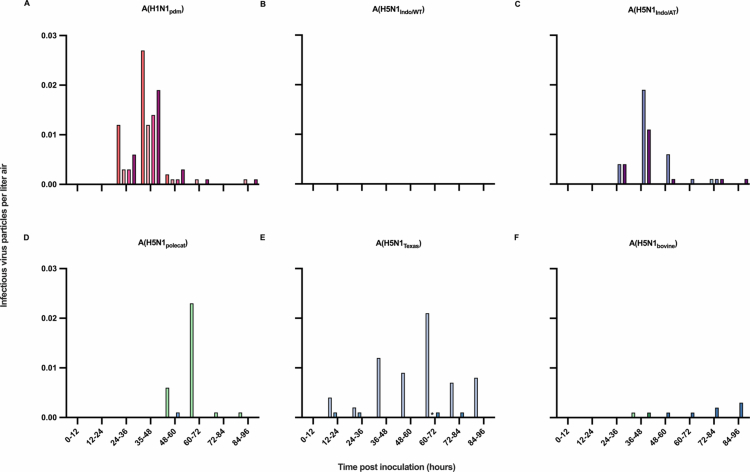
Infectious virus particles per liter air. **A**) A(H1N1_pdm_), **B**) A(H5N1_Indo/WT_), **C**) A(H5N1_Indo/AT_), **D**) A(H5N1_polecat_), **E**) A(H5N1_Texas_), **F**) A(H5N1_bovine_). Each differently colored bar represents an individual ferret (four ferrets per virus). *This animal was euthanized at 72 hpi because of reaching predetermined humane endpoints, thus no air-samples were collected after the 72 hpi timepoint. Source data

To address these questions and to gain a deeper understanding of the risks associated with mammalian-transmissible A(H5N1) viruses, we here extend beyond conventional ferret transmission studies. We designed an experimental set-up in which a cage with an infected donor animal was directly connected to a BioSpot-VIVAS series 315 Bioaerosol Sampler (Extended Data Fig. 1), to allow continuous sampling of infectious viruses expelled in the air. Sequential 12 h air samples were collected from four individually housed ferrets per virus. Initial experiments were conducted with the 2009 pandemic A(H1N1_pdm_) virus A/Netherlands/602/2009, which previously transmitted via the air in four out of four donor–recipient pairs (Table 1)^7^. All four A(H1N1_pdm_)-inoculated animals shed infectious virus by 24 hours post infection (hpi), which peaked around 36–48 hpi with approximately 200 infectious virus particles being collected (Fig. 1a and Extended Data Fig. 2). This peak virus shedding at day 2 is consistent with other studies where either infectious A(H1N1_pdm_)^8^ or RNA^9,10^ was detected in air samples.

**Table 1:** Transmission and virus shedding in experimentally infected animals

Influenza virus	Transmission efficiency between ferrets as demonstrated by virus isolation from recipient	Infectious virus collected from air (this study)
A(H1N1pdm)	4/4 (ref. ^30^)	4/4
A(H5N1_Indo/WT_)	0/4 (ref. ^7^)	0/4
A(H5N1_Indo/AT_)	3/4 (ref. ^11^)	2/4
A(H5N1_polecat_)	1/4^$^	1/4
A(H5N1_Texas_)	10/30 (refs. ^14,18^)	1/4
A(H5N1_bovine_)	0/4* (ref. ^19^)	0/4

*Seroconversion was demonstrated in one animal.

**Extended Data Fig. 3:**
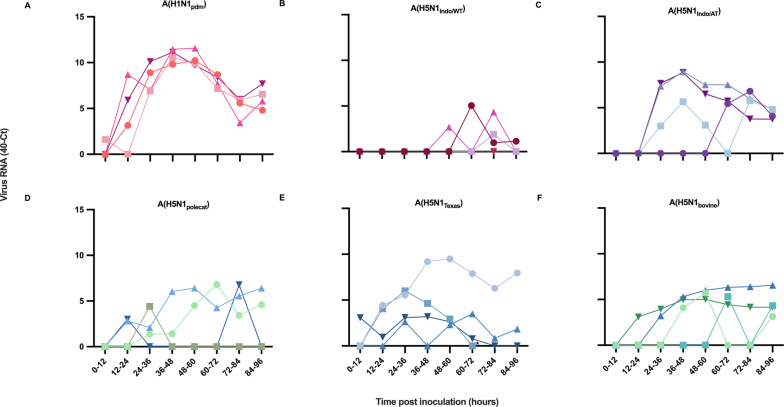
Viral RNA in air-samples. **A**) A(H1N1_pdm_), **B**) A(H5N1_Indo/WT_), **C**) A(H5N1_Indo/AT_), **D**) A(H5N1_polecat_), **E**) A(H5N1_Texas_), **F**) A(H5N1_bovine_). Each symbol represents an individual ferret (four ferrets per virus) using the same colors as in Fig. 1. RNA was detected by qRT-PCR. *This animal was euthanized at 72 hpi because of predetermined humane endpoints, thus no air-samples were collected after the 72 hpi timepoint. Source data

**Extended Data Fig. 4:**
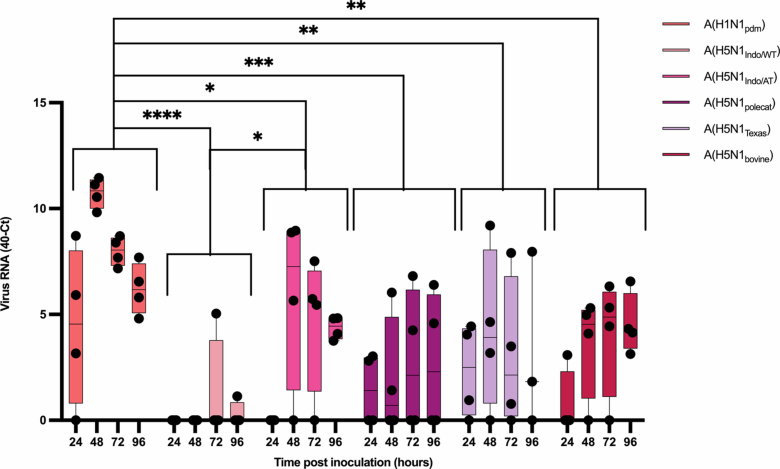
Comparison of viral RNA load in air-samples across different viruses. Four biological replicates were performed and are derived from individual ferrets, each point in the box plots represents an individual ferret. The box plots contain the 5th to 95th percentile, the whiskers mark the minimum and the maximum, and the black line in the middle marks the mean of the dataset. Statistical significance was assessed using a two-way ANOVA with Tukey’s multiple comparison test. *p = 0.02; **p = 0.004; ***p = 0.0005; ****p = 0.00002. Source data

Next, zoonotic A(H5N1) virus A/Indonesia/5/2005 (A(H5N1_Indo/WT_)) and a modified version of this virus that was previously found to be transmissible via the air between ferrets (A(H5N1_Indo/AT_)) were tested^11^. A(H5N1_Indo/AT_) harbours six mammalian adaptation substitutions resulting in a shift in binding of the influenza virus haemagglutinin (HA) surface protein from avian-type α-2,3- to human-type α-2,6-linked sialic acid receptors, an increased HA acid stability and enhanced genome replication in mammalian cells^11^. (A(H5N1_Indo/WT_) and A(H5N1_Indo/AT_) were previously found to transmit in zero out of four and three out of four donor–recipient pairs, respectively (Table 1)^11^. However, it was still unknown whether this difference was due to inefficient shedding of infectious virus into the air or the absence of necessary phenotypic properties to initiate an infection in a new host. Here, we found that ferrets inoculated with non-mammal-adapted A(H5N1_Indo/WT_), did not shed infectious viruses in the air (Fig. 1b). By contrast, infectious virus was captured from the cages of two out of four A(H5N1_Indo/AT_)-inoculated animals, with peak virus shedding of 207 and 66 infectious virus particles between 36 hpi and 48 hpi (Fig. 1c). The maximum amount of A(H5N1_Indo/WT_) RNA detected in air samples was approximately 200-fold and 650-fold lower than levels observed for A(H5N1_Indo/AT_) and A(H1N1_pdm_), respectively (Extended Data Fig. 3). The viral RNA levels in the air of A(H5N1_Indo/AT_) and A(H5N1_Indo/WT_) were significantly different (*P* = 0.02; Extended Data Fig. 4), indicating that A(H5N1_Indo/WT_) is not transmitted due to its inefficient expulsion into the air, rather than to mechanisms acting in the environment or in the recipient. This aligns with a previous study, which showed that airborne-transmissible viruses exhibit high replication in the upper respiratory tract and fast release in the air compared with non-transmissible viruses that show lower viral RNA levels in air sampled from infected ferrets^12^.

Interestingly, whereas in older studies A(H5) viruses such as A(H5N1_Indo/WT_) were never transmitted via the air between ferrets, low levels of transmission have lately been observed for recently emerged clade 2.3.4.4b A(H5) viruses isolated from a European polecat, mink and dairy cows^13,14^. These observations raise concerns regarding the evolution of an A(H5N1) towards mammalian, and possibly human, adaptation. Therefore, we next tested influenza A(H5N1) clade 2.3.4.4b viruses collected from mammals during the recent global epizootic.

In 2022, we isolated an A(H5N1) virus from a European polecat^15^, A/European polecat/Netherlands/1/2022 (A(H5N1_polecat_)), that harbours the mammalian adaptation substitution T271A in the polymerase basic protein 2 (PB2) of the polymerase complex^16,17^. This virus was previously transmitted via the air to one out of four recipient animals (Table 1), which corresponds with the virus shedding by only one animal, which started after 48 hpi and peaked at 60–72 hpi (243 infectious virus particles; Fig. 1d).

Lastly, we evaluated the virus shedding kinetics of two A(H5N1) viruses isolated during the ongoing outbreak in US cattle: A/Texas/37/2024 (A(H5N1_Texas_)) with the mammalian adaptation substitution E627K in PB2, isolated from a dairy farm worker, and A/Bovine/Ohio/B24OSU-439/2024 (A(H5N1_bovine_), from an outbreak in dairy cattle in Ohio. Whereas A(H5N1_bovine_) was recently found to not be transmissible via the air between ferrets, A(H5N1_Texas_) was reported to be transmitted to 10 out of 30 recipient animals across two combined studies^14,18^. In agreement with the reported transmission data, only one of the A(H5N1_Texas_)-inoculated animals continuously shed infectious virus in the air from 12 hpi onwards (Fig. 1e). Virus shedding peaked at 60–72 hpi, similar to the kinetics found for A(H5N1_polecat_). No noteworthy infectious virus shedding was observed for the A(H5N1_bovine_), which also aligns with the previously reported results^19^, except for low shedding in the air by one animal at the last sampling timepoint (30 infectious virus particles at 84–96 hpi) (Fig. 1f).

Although it is uncertain whether we capture every single expelled infectious virus particle from the air in our experimental set-up, or only a fraction of it, our data reveal that the virus shedding kinetics, as measured through air sampling, bear a remarkable resemblance to data on outcomes of ferret-to-ferret transmission studies and provide new insights into the dynamics of influenza A virus transmission (Table 1). In our studies, only for the human A(H1N1_pdm_) virus, infectious virus was found in the air for all ferrets. Interestingly, A(H1N1_pdm_) was also the virus for which significantly more viral RNA was collected from the air as compared with all other viruses tested, indicating that the viral RNA load in the air may be a proxy for transmission efficiency (Extended Data Fig. 4).

**Extended Data Fig. 5:**
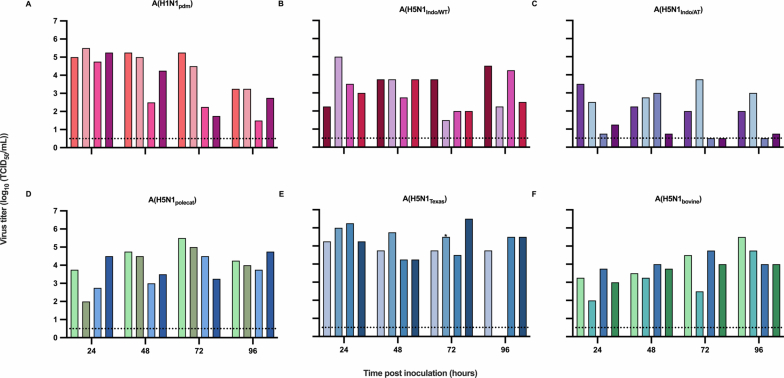
Infection virus titers in ferret throat swabs. **A**) A(H1N1_pdm_), **B**) A(H5N1_Indo/WT_), **C**) A(H5N1_Indo/AT_), **D**) A(H5N1_polecat_), **E**) A(H5N1_Texas_), **F**) A(H5N1_bovine_). Each differently colored bar represents a single ferret (four ferrets per virus). Dotted horizontal lines represent the detection limit. *This animal was euthanized at 72 hpi because of predetermined humane endpoints, thus no throat swabs were collected after the 72 hpi timepoint. Source data

**Extended Data Fig. 6:**
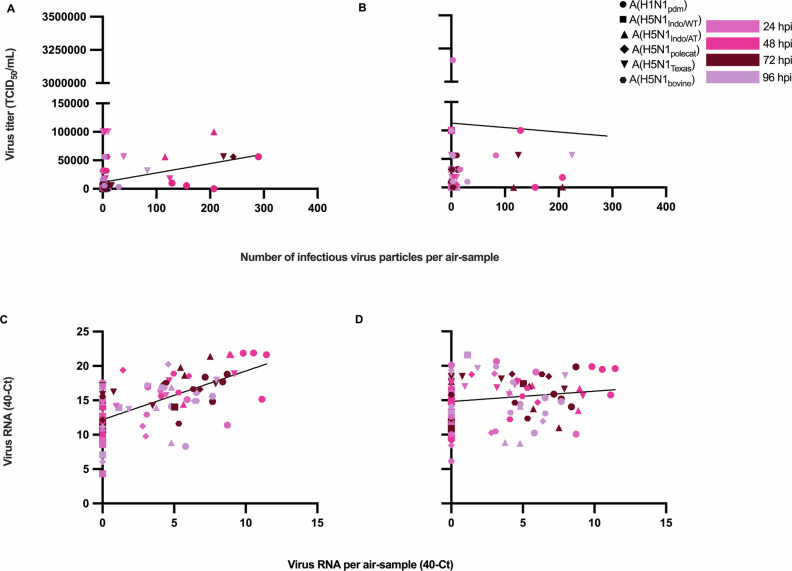
Correlation analyses of the presence of infectious virus or viral RNA in ferret nose and throat swabs and air-samples. Correlation between number of infectious virus in air-sample and infectious virus titer in **A**) nose swabs (R^2^ = 0,1256), and **B**) throat swabs (R^2^ = 0,0001). Correlation between amount of viral RNA in air-sample and viral RNA in **C**) nose swabs (R^2^ = 0.3789), and **D**) throat swabs (R2 = 0.0222). Each symbol represents one of the six viruses tested. Each differently colored symbol represents a time point post inoculation in hours (hpi). The statistical analysis performed is simple linear regression. Source data

We recently demonstrated that influenza A viruses are primarily transmitted from the nasal respiratory epithelium^20^. Ferrets with higher levels of infectious virus in the nose also tended to shed more virus into the air, with peak virus shedding in the air seen in ferrets with the highest nasal titres on peak shedding days (Fig. 1g–l). Notably, ferrets 3 and 4 in the A(H5N1_Indo/AT_) group, ferret 1 in the A(H5N1_polecat_) group and ferret 1 in the A(H5N1_Texas_) group exhibited the highest virus titres in nasal swabs (indicated by symbols in Fig. 1). However, no significant correlation was found between the amount of infectious virus or viral RNA expelled in the air and the virus titres in the nose or throat swabs (Extended Data Figs. 5 and 6).

The virus shedding by one A(H5N1_polecat_)- and one A(H5N1_Texas_)-inoculated animal and the low transmission efficiency of these viruses reported by others^14,18^ may be due to mammalian adaptation substitutions in the PB2 protein. A(H5N1_Texas_) harbours PB2-E627K, whereas A(H5N1_polecat_) and the A(H5N1) mink virus that were found to be transmissible between ferrets contain PB2 T271A^13^. Additional studies should investigate if the potentially higher replication due to these PB2 substitutions is enough to allow low levels of transmission via the air, despite the preferential binding to avian-type receptors and acid instability of HA.

Our results indicate that recent A(H5N1) viruses exhibit a low but increased level of infectious virus shedding into the air as compared with older A(H5N1) viruses. Given the ongoing epizootic in cattle and the high risk of exposure for farm and dairy workers and domestic and wild mammals to infected cows and contaminated milk, it is crucial for effective outbreak control and public health safety to understand how this virus spreads among cattle, its potential for mammalian adaptation and its capacity for airborne transmission.

## Methods

### Ethics statement

Animals were housed and experiments were performed in strict compliance with the Dutch legislation for the protection of animals used for scientific purposes (2014, implementing EU Directive 2010/63). Twenty-four influenza virus and Aleutian disease virus-seronegative 1–2-year-old female ferrets (*Mustela putorius furo*), weighing 575–965 g, were obtained from a commercial breeder (TripleF). The studies were performed under a project licence from the Dutch competent authority Centrale Commissie Dierproeven (licence number CCD101002115685) and the study protocols were approved by the institutional Animal Welfare Body (Erasmus MC permit number 2400041). Animal welfare was monitored on a daily basis.

### Biosafety

Experiments with A(H1N1pdm) were performed under animal biosafety level 3 (ABSL 3) conditions, and experiments with A(H5N1_Indo/WT_), A(H5N1_polecat_), A(H5N1_Indo/AT_), A(H5N1_Texas_), and A(H5N1_bovine_) were performed under enhanced ABSL 3+ conditions. The ABSL3+ facility of Erasmus MC consists of a negative pressurized (30 Pa) laboratory in which all in vivo and in vitro experimental work is carried out in class 3 isolators or class 3 biosafety cabinets, which are also negative pressurized (≤200 Pa). Although the laboratory is considered ‘clean’ because all experiments are conducted in closed class 3 cabinets and isolators, special personal protective equipment, including laboratory suits, gloves and FFP3 facemasks, is used. Air released from the class 3 units is filtered by high-efficiency particulate air filters and leaves the facility via a second set of high-efficiency particulate air filters. Only authorized personnel that received the appropriate training can access the ABSL3+ facility. All personnel working in the facility is vaccinated against seasonal influenza viruses. For animal handling in the facilities, personnel always work in pairs. The facility is secured by procedures recognized as appropriate by the institutional biosafety officers and facility management at Erasmus MC, Dutch and US government inspectors. Antiviral drugs (oseltamivir and zanamivir) and personnel isolation facilities are directly available to further mitigate risks upon incidents.

### Cells

Madin–Darby canine kidney (MDCK) cells (ATCC-CRL-2935) as well as humanized MDCK cells (hCK)^21^ were cultured at 37 °C, 5% CO_2_ in Minimum Essential Medium (MEM) Eagle with Earle’s balanced salt solution (Capricorn Scientific) supplemented with 10% foetal bovine serum (Sigma-Aldrich), 10 mM HEPES (Capricorn Scientific), 100 lU ml^−1^ penicillin (PEN; Capricorn Scientific), 100 µg ml^−1^ streptomycin (STR; Capricorn Scientific), 1× MEM non-essential amino acids (NEAA; Capricorn Scientific), 2 mM l-glutamine (l-glu; Capricorn Scientific) and 1.5 mg ml^−1^ sodium bicarbonate (NaHCO_3_; Gibco). In addition, hCK cells were cultured with 2 µg ml^−1^ puromycin (InvivoGen) and 10 µg ml^−1^ blasticidin (InvivoGen). 293T cells (ATCC-CRL-3216) were cultured in Dulbecco’s modified Eagle medium, high glucose (4.5 g l^−1^) (Capricorn Scientific) supplemented with 10% foetal bovine serum, 100 lU ml^−1^ PEN, 100 µg ml^−1^ STR, 2 mM l-glu, 1 mM sodium pyruvate (Gibco) and 1× NEAA.

### Viruses

Recombinant A/Netherlands/602/2009 (A(H1N1pdm), GISAID accession numbers EPI178246-250, EPI178467, EPI178290 and EPI178291), A/Indonesia/5/2005 wild type (A(H5N1_Indo/WT_), EPI376534-EPI376541) and airborne transmissible (AT) (A(H5N1_Indo/AT_) containing PB2-E627K, PB1-H99Y, HA-H103Y, HA-T156A, HA-Q222L and HA-G224S), A/European polecat/Netherlands/1/2022 (A(H5N1_polecat_), EPI2061044-EPI2061051) and A/Texas/37/2024 (A(H5N1_Texas_), EPI3171486- EPI3171493) were generated using the eight-plasmid rescue system as previously described^22^. Influenza virus isolate A/Bovine/Ohio/B24OSU-439/2024 (A(H5N1_bovine_), EPI3352841-EPI3352848) was passaged once in embryonated eggs and MDCK cells.

### Ferret air-sampling experiment

Four ferrets per group were inoculated intranasally with 10^6^ 50% tissue culture infectious dose (TCID_50_) of virus diluted in 500 µl phosphate-buffered saline. A total of 250 µl of virus was instilled dropwise in each ferret nostril. Inoculations were done under anaesthesia with a mixture of ketamine and medetomidine (10 mg kg^−1^ and 0.05 mg kg^−1^, respectively) antagonized by atipamezole (0.25 mg kg^−1^). Ferret throat and nose swabs were collected daily under light anaesthesia using ketamine to minimize animal suffering. After collection, swabs (cat. no. 155CS01, Copan) were stored at −80 °C in virus transport medium consisting of MEM with Hanks’ BSS (Gibco), containing 0.5% lactalbumin hydrolysate (Sigma-Aldrich), 10% glycerol (Sigma-Aldrich), 200 lU ml^−1^ PEN, 200 mg ml^−1^ STR, 10 MU polymyxin B sulfate (Sigma-Aldrich), 5 MU nystatin (Sigma-Aldrich) and 250 mg ml^−1^ gentamicin (Gibco). Before their storage, 60 µl of each throat or nose swab were taken and mixed with 90 µl of MagNA Pure 96 External Lysis Buffer (Roche Diagnostics) for RNA isolation.

For the collection of air samples, the BioSpot-VIVAS Series 315 bioaerosol sampler (Aerosol Devices) was connected directly to a ferret cage holding an individually housed ferret, via a 1.4-m-long tubing. The BioSpot was operated with a flowrate of 15 l min^−1^ under the following conditions: conditioner: 5 °C; initiator: 45 °C; moderator: 18 °C; nozzle: 27 °C; sample holder: between 13 °C and 16 °C. No extra airflow was supplied to the ferret cage, in addition to the 15 l min^−1^ applied by the BioSpot sampler. With a cage volume of 45 l (30 × 30 × 50 cm) and a flow rate of 900 l h^−1^ (60 × 15 l min^−1^), this results in 20 (900 l h^−1^/45 l) air changes per hour. The ferret cage and BioSpot were housed in separate, interconnected, negatively pressurized class 3 isolators (Extended Data Fig. 1). This separation was necessary because the BioSpot generated excessive heat, which led to an increased ambient temperature within the isolator. Air sampling was conducted over a 96 h period with continuous sampling and collection of samples every 12 h. The average temperature and relative humidity in the isolators with the ferret cages were 22 °C and 51%, respectively. Air samples were collected in a Petri dish (Falcon, 35 mm) containing 2 ml of virus transport medium. The samples were stored at 4 °C until end-point titration, performed directly the following day to prevent freeze–thaw cycles.

### Quantification of infectious virus in air samples

The volume of the collected air samples ranged from 2 to 4 ml, and from each sample 60 µl was taken and mixed with 90 µl of MagNA Pure 96 External Lysis Buffer (Roche) for subsequent RNA isolation. The volume of the sample was then adjusted to a total of 6 ml (referred to as undiluted sample) with virus infection medium: MEM with Earle’s BSS supplemented with 100 lU ml^−1^ PEN, 100 µg ml^−1^ STR, 2 mM l-glu, 1× NEAA, 1.5 mg ml^−1^ NaHCO_3_, 10 mM HEPES and 0.8 µg ml^−1^ tosyl phenylalanyl chloromethyl ketone-treated trypsin (Sigma-Aldrich). From the undiluted sample, 500 µl was taken and 10-fold diluted in 4.5 ml of infection medium. hCK cells were seeded on a 96-well cell culture plate the day before. The entire volume of the air sample was used for the inoculation of cells. To half of the 96-well plate, 100 µl of either the undiluted or the 10-fold diluted sample was added and cells were incubated with the inoculum for 2 h at 37 °C, 5% CO_2_. After the incubation, the inoculum was removed and 200 µl of infection medium was added to each well, followed by incubation for 3–5 days at 37 °C, 5% CO_2_, after which supernatants of cell cultures were tested for agglutination activity using 0.3% turkey erythrocytes to determine the number of positive wells per air sample. The number of infectious virus particles per air sample was calculated using a positive well correction method^23^.

### Virus titrations

Virus stocks, throat and nose swabs were titrated on MDCK cells. Briefly, MDCK cells were inoculated with 10-fold serial dilutions of each sample in infection medium. Infectious virus titres (TCID_500_ ml^−1^) were calculated from ten replicates for each of the virus stocks or four replicates for each throat and nose swab, using the Spearman–Karber method^24,25^.

### RNA isolation and real-time qRT–PCR

RNA isolation from the air samples, throat and nose swabs was performed using magnetic beads (AMPure XP, Beckman Coulter)^26^. After isolation, the RNA was kept on an ice block and 8 µl of it was transferred to a new plate with quantitative reverse transcription polymerase chain reaction (qRT–PCR) mix, containing 0.4 µl of primers and probe mix targeting the M gene of influenza A viruses^27^, 0.4 µl of primers and probe mix targeting the HA gene of phocine distemper virus (as an internal control for the RNA extraction)^28^, as well as 4 µl of TaqMan Fast Virus 1-step master mix (ThermoFisher Scientific) and 6.2 µl of PCR-grade water. The cut-off threshold was set manually after examination of the background signals and the negative control. Samples with a cycle threshold (Ct) of 40 and above were considered negative. Here, the qRT–PCR data are represented as 40-Ct. The following primers and probes were used: influenza A virus: 5′-CTTCTRACCGAGGTCGAAACGTA-3′ (forward), 5′-TCTTGTCTTTAGCCAYTCCATGAG-3′ (reverse), probe 1 5′-FAM(6-carboxyfluorescein)-TCAGGCCCCCTCAAAGCCGAGA-BHQ(black hole quencher)-3′, probe 2 5′-FAM-TCAGGCCCCCTCAAAGCCGAAA-BHQ-3′; phocine distemper virus: 5′-CGGGTGCCTTTTACAAGAAC-3′ (forward), 5′-TTCTTTCCTCAACCTCGTCC-3′ (reverse), probe 5′-Cy5-ATGCAAGGGCCAATT-MGB(minor groove binder)-Eclipse-3′. The amplification and detection were performed on an ABI7700 (Thermo Fisher Scientific) with the following cycler parameters: 5 min 50 °C, 20 s 95 °C, (3 s 95 °C, 31 s 60 °C) × 45 cycles.

### Multi-segment RT–PCR and whole-genome sequencing

To determine the whole-genome consensus sequence of the viruses used in the ferret studies, before ferret inoculation, RNA was extracted from the virus stocks using the High Pure RNA Isolation Kit (Roche), according to the manufacturer’s instructions. A multi-segment reverse transcription polymerase chain reaction (RT–PCR) was performed using SuperScript III One-Step RT–PCR System with Platinum Taq High Fidelity DNA Polymerase (Invitrogen). The primers used for amplification of the virus genome were the following: 5′-TGTACTACTCAGCRAAAGCAGG-3′ (forward), and 5′-TGTACTACTCAGTAGAAACAAGG-3′ (reverse). The cycler settings used were as follows: 2 min 55 °C, 60 min 42 °C, 2 min 94 °C, (30 s 94 °C, 30 s 44 °C, 3.5 min 68 °C) × 5 cycles, (30 s 94 °C, 30 s 57 °C, 3.5 min 68 °C) × 35 cycles, 10 min 68 °C. Libraries were generated using a ligation sequencing kit (SQK-LSK114, native barcoding kit, Oxford Nanopore Technologies) and were multiplexed and sequenced on a MinION R10 flowcell (Oxford Nanopore Technologies) according to the manufacturer’s instructions. Data analysis was performed using the Iterative Refinement Meta-Assembler (IRMA)^29^ version V1.1.4, using the FLU-minion module with the following configuration changes: ‘SKIP_E = 0’, ‘RESIDUAL_ASSEMBLY_FACTOR = 100’, ‘DO_SECONDARY = 1’ and ‘MIN_LEN = 700’.

### Reporting summary

Further information on research design is available in the Nature Portfolio Reporting Summary linked to this article.

## Supplementary information

Reporting Summary

## Source data

Source Data Fig. 1 Raw data air-sample quantification and ferret nose swabs titrations.

Source Data Extended Data Fig.2 Raw data air-sample quantification.

Source Data Extended Data Fig.3 TaqMan data of air samples per virus (40-Ct).

Source Data Extended Data Fig.4 TaqMan data of air samples (40-Ct).

Source Data Extended Data Fig.5 Ferret throat swab titrations.

Source Data Extended Data Fig.6 Raw data air-sample number of infectious virus particles per sample and ferret nose or throat swab titrations. TaqMan data for air samples and nose or throat swabs (40-Ct).

## Data Availability

Source data are provided with this paper.
