# Supplementary material for: Influenza A(H5N1) shedding in air corresponds to transmissibility in mammals
Source: Nat Microbiol. 2024 Dec 2;10(1):14–9. doi: 10.1038/s41564-024-01885-6 (PMC11726459; doi:10.1038/s41564-024-01885-6)
Supplement: Supplementary file 1 — Reporting Summary [file 41564_2024_1885_MOESM1_ESM.pdf]

nature portfolio

Corresponding author(s): Sander Herfst

Last updated by author(s): Nov 4, 2024

Reporting Summary

Nature Portfolio wishes to improve the reproducibility of the work that we publish. This form provides structure for consistency and transparency in reporting. For further information on Nature Portfolio policies, see our Editorial Policies and the Editorial Policy Checklist.

Statistics

For all statistical analyses, confirm that the following items are present in the figure legend, table legend, main text, or Methods section.

n/a	Confirmed	
☐	☒	The exact sample size (*n*) for each experimental group/condition, given as a discrete number and unit of measurement
☒	☐	A statement on whether measurements were taken from distinct samples or whether the same sample was measured repeatedly
☐	☒	The statistical test(s) used AND whether they are one- or two-sided<br>*Only common tests should be described solely by name; describe more complex techniques in the Methods section.*
☒	☐	A description of all covariates tested
☒	☐	A description of any assumptions or corrections, such as tests of normality and adjustment for multiple comparisons
☐	☒	A full description of the statistical parameters including central tendency (e.g. means) or other basic estimates (e.g. regression coefficient) AND variation (e.g. standard deviation) or associated estimates of uncertainty (e.g. confidence intervals)
☐	☒	For null hypothesis testing, the test statistic (e.g. *F*, *t*, *r*) with confidence intervals, effect sizes, degrees of freedom and *P* value noted<br>*Give P values as exact values whenever suitable.*
☒	☐	For Bayesian analysis, information on the choice of priors and Markov chain Monte Carlo settings
☒	☐	For hierarchical and complex designs, identification of the appropriate level for tests and full reporting of outcomes
☒	☐	Estimates of effect sizes (e.g. Cohen's *d*, Pearson's *r*), indicating how they were calculated

Our web collection on statistics for biologists contains articles on many of the points above.

Software and code

Policy information about availability of computer code

Data collection	No software was used.
Data analysis	For the analysis of the Minion (Oxford Nanopore technologies) sequences of the virus stocks generated for ferret inoculations, the iterative refinement meta-assembler software (IRMA) was used. The IRMA version that was used is the following: v.1.1.4, with module "FLU-minion".

For manuscripts utilizing custom algorithms or software that are central to the research but not yet described in published literature, software must be made available to editors and reviewers. We strongly encourage code deposition in a community repository (e.g. GitHub). See the Nature Portfolio guidelines for submitting code & software for further information.

Data

Policy information about availability of data

All manuscripts must include a data availability statement. This statement should provide the following information, where applicable:

- Accession codes, unique identifiers, or web links for publicly available datasets
- A description of any restrictions on data availability
- For clinical datasets or third party data, please ensure that the statement adheres to our policy

Source data are provided with this paper

Research involving human participants, their data, or biological material

Policy information about studies with human participants or human data. See also policy information about sex, gender (identity/presentation), and sexual orientation and race, ethnicity and racism.

Reporting on sex and gender	Not applicable
Reporting on race, ethnicity, or other socially relevant groupings	Not applicable
Population characteristics	Not applicable
Recruitment	Not applicable
Ethics oversight	Not applicable

Note that full information on the approval of the study protocol must also be provided in the manuscript.

Field-specific reporting

Please select the one below that is the best fit for your research. If you are not sure, read the appropriate sections before making your selection.

☒ Life sciences ☐ Behavioural & social sciences ☐ Ecological, evolutionary & environmental sciences

For a reference copy of the document with all sections, see nature.com/documents/nr-reporting-summary-flat.pdf

Life sciences study design

All studies must disclose on these points even when the disclosure is negative.

Sample size	The samples size used in this study was 4 animals (ferrets) per virus group. This was based on previously published work on influenza virus and SARS-CoV-2 transmission studies and this groupsize is commonly used by others as well (e.g. PMID: 35862762 (Robustness of the Ferret Model for Influenza Risk Assessment Studies: a Cross-Laboratory Exercise), PMID: 32641684 (SARS-CoV-2 is transmitted via contact and via the air between ferrets)).No statistical analysis were used to predefine the sample size, this is due to that fact that the researcher is required to keep the effects of the intervention to a minimum. This is based on the three R system for animal experimentation, meaning Refinement, Replacement, and Reduction.
Data exclusions	No data were excluded from the analysis.
Replication	The experimental set-up was previously verified with another set of viruses and compared to previous set-ups, in order to ensure that we are able to collect virus particles at a constant rate and also for different influenza A viruses. The ferret air-sampling experiments were performed once, with four ferrets per virus. Next, the use of specific cell line for the quantification of virus particles from the air-samples that has higher sensitivity compared to conventional cell lines used for influenza virus research allows for the better and more reproducible quantification of the amount of infections virus particles. For the quantification of the air-samples no replicates were done, as the entire samples was used for the hCK infection. The titrations of ferret throat and nose swabs were done in quadruplicates.
Randomization	The animals were allocated to the different groups randomly.
Blinding	The investigators in this study were not blinded. This was not possible because regulations require that the different viruses were handled separately.

Reporting for specific materials, systems and methods

We require information from authors about some types of materials, experimental systems and methods used in many studies. Here, indicate whether each material, system or method listed is relevant to your study. If you are not sure if a list item applies to your research, read the appropriate section before selecting a response.

Materials & experimental systems

n/a	Involved in the study	
☒	☐	Antibodies
☐	☒	Eukaryotic cell lines
☒	☐	Palaeontology and archaeology
☐	☒	Animals and other organisms
☒	☐	Clinical data
☐	☒	Dual use research of concern
☒	☐	Plants

Methods

n/a	Involved in the study	
☒	☐	ChIP-seq
☒	☐	Flow cytometry
☒	☐	MRI-based neuroimaging

Eukaryotic cell lines

Policy information about cell lines and Sex and Gender in Research

Cell line source(s)	MDCK cells were acquired from ATCC, hCK cells were obtained from Prof. Kawaoka, 293T cells were acquired from ATCC.
Authentication	None of the cell lines used were authenticated.
Mycoplasma contamination	All cell lines tested negative for mycoplasma contamination.
Commonly misidentified lines (See ICLAC register)	None

Animals and other research organisms

Policy information about studies involving animals; ARRIVE guidelines recommended for reporting animal research, and Sex and Gender in Research

Laboratory animals	1-2-years-old female ferret (Mustela putorius furo) were used. Their weight was 575-965g.
Wild animals	No wild animals were used in this study.
Reporting on sex	For this study female ferret were used and their ability to shed infectious virus in the air was examined. we did not expect any inter-sex differences between the amount of virus shed in the air, therefore our findings apply not only for female animals but also male.
Field-collected samples	No field-collected samples were used in this study.
Ethics oversight	The animals were house and the experiments were performed ynder strict cimpliance to the Dutch legislation for the protection of animals used for scientific purposes (2014, implementing EU Directive 2010/63). The studies were performed under a project license from the Dutch competent authority Centrale Commissie Dierproeven (license number CCD101002115685) and the study protocols were approved by the institutional Animal Welfare Body (Erasmus MC permit number 2400041).

Note that full information on the approval of the study protocol must also be provided in the manuscript.

Dual use research of concern

Policy information about dual use research of concern

Hazards

Could the accidental, deliberate or reckless misuse of agents or technologies generated in the work, or the application of information presented in the manuscript, pose a threat to:

No	Yes	
☐	☒	Public health
☒	☐	National security
☒	☐	Crops and/or livestock
☒	☐	Ecosystems
☒	☐	Any other significant area

Hazards	A previously generated A(H5N1) virus that was transmissible via the air between ferrets was used in these studies (PMID: 22723413), so no new dual use research of concern is presented here.

For examples of agents subject to oversight, see the United States Government Policy for Institutional Oversight of Life Sciences Dual Use Research of Concern.

Experiments of concern

Does the work involve any of these experiments of concern:

No	Yes	
☒	☐	Demonstrate how to render a vaccine ineffective
☒	☐	Confer resistance to therapeutically useful antibiotics or antiviral agents
☒	☐	Enhance the virulence of a pathogen or render a nonpathogen virulent
☒	☐	Increase transmissibility of a pathogen
☒	☐	Alter the host range of a pathogen
☒	☐	Enable evasion of diagnostic/detection modalities
☒	☐	Enable the weaponization of a biological agent or toxin
☒	☐	Any other potentially harmful combination of experiments and agents

Precautions and benefits

Biosecurity precautions	*Describe the precautions that were taken during the design and conduct of this research, or will be required in the communication and application of the research, to minimise biosecurity risks. These may include bio-containment facilities, changes to the study design/ methodology or redaction of details from the manuscript.*
Biosecurity oversight	*Describe any evaluations and oversight of biosecurity risks of this work that you have received from people or organizations outside of your immediate team.*
Benefits	*Describe the benefits that application or use of this work could bring, including benefits that may mitigate risks to public health, national security, or the health of crops, livestock or the environment.*
Communication benefits	*Describe whether the benefits of communicating this information outweigh the risks, and if so, how.*

Plants

Seed stocks	Not applicable
Novel plant genotypes	Not applicable
Authentication	Not applicable
